# Dynamic Model for the Epidemiology of Diarrhea and Simulation Considering Multiple Disease Carriers

**DOI:** 10.3390/ijerph17165692

**Published:** 2020-08-06

**Authors:** Firda Rahmadani, Hyunsoo Lee

**Affiliations:** School of Industrial Engineering, Kumoh National Institute of Technology, Gumi, Gyeongbuk 39177, Korea; firdarahmadani13@kumoh.ac.kr

**Keywords:** dynamic epidemiology, multiple disease carriers, diarrhea, infection process-based dynamic control, Pontryagin’s maximum principle

## Abstract

Diarrhea is responsible for killing around 525,000 children every year, even though it is preventable and treatable. This research focuses on both houseflies’ roles and humans’ roles in carrying pathogens causing diarrhea as multiple disease carriers. Both human and fly compartmental models are simulated with five diseases control strategies in order to identify the epidemic dynamics. The framework considers the life cycle of flies modeled into eggs, larvae, pupae, susceptible flies, and carrier flies, while the human system follows a compartment model as susceptible, infected, recovered, and back to susceptible again (SIRS). The relationships are modeled into an ordinary differential equation-based compartmental system. Then, the control parameters of the compartmental framework are analyzed. In order to propose effective control methods, five control strategies are considered: (1) elimination of flies’ breeding site, (2) sanitation, (3) installation of UV light trap, (4) good personal and food hygiene, and (5) water purification. Then, overall, ten control scenarios using the five control strategies are analyzed. Among them, effective control solutions considering various dynamic epidemiology are provided with the simulations and analyses. The proposed framework contributes to an effective control strategy in reducing the number of both flies and infected humans, since it minimizes the spread of the disease and considers cost-effectiveness.

## 1. Introduction

Infectious diarrhea, a disease causing fluid loss and dehydration, is the eighth leading cause of death, responsible for around 525,000 children deaths globally every year [1], mostly children in developing countries. Diarrhea is caused by infectious organisms, including viral and bacterial pathogens [2]. These are typically transmitted from the stool of one individual. This means the pathogen spreads through contaminated food and water or from an infectious person to a healthy person as a result of poor hygiene. Although diarrhea is both preventable and treatable, it is still fatal. This is due to several reasons, including a lack of awareness and shortages of existing lifesaving interventions.

Several existing studies showed that bacteria causing diarrhea are carried by houseflies [3,4] mainly. The house fly, *Musca domestica L.*, is known to carry pathogens including bacteria, viruses, fungi, and parasites which cause life threatening diseases in humans and animals [5,6]. Houseflies breed in human feces [7] and the existing studies [8,9,10,11,12] have shown increased incidences of diarrhea during the periods of high fly density. Most importantly, several existing studies [8,13,14,15] have shown that a reduction in housefly density may affect the reduction in diarrhea incidence as well.

Mathematical models [16] are widely used to convert the real case into mathematical epidemiologic representations and predict the dynamics of infectious disease transmission so that they have vital roles in developing public health strategies for disease control and prevention. The formulation process of diarrhea considers multiple carriers (e.g., houseflies and infected humans). Then, the mathematical model is used for relevant disease control. Effective disease control has become an important part of computational epidemiology [17] that can provide useful guidelines for designing effective disease intervention strategies while balancing the costs of the control measures.

The objective of this paper is to understand and to formulate diarrhea’s dynamic epidemiology through a set of differential equation-based mathematical models and to predict the possible future for the effectiveness of disease control strategies not only for reducing the infected population, but also decreasing or even eliminating fly population as principal carriers of the disease as well.

The following section presents the relevant epidemiologic network of diarrhea and its mathematical models along with multiple controls and their theoretical solutions. Then, the effectiveness of the proposed framework and simulation results are analyzed in Section 3 and Section 4. In order to show the influence of each intervention towards the spreading of the disease, numerical simulations under several control scenarios are provided in Section 5.

## 2. Background and Epidemiologic Network Model

**Figure 1:**
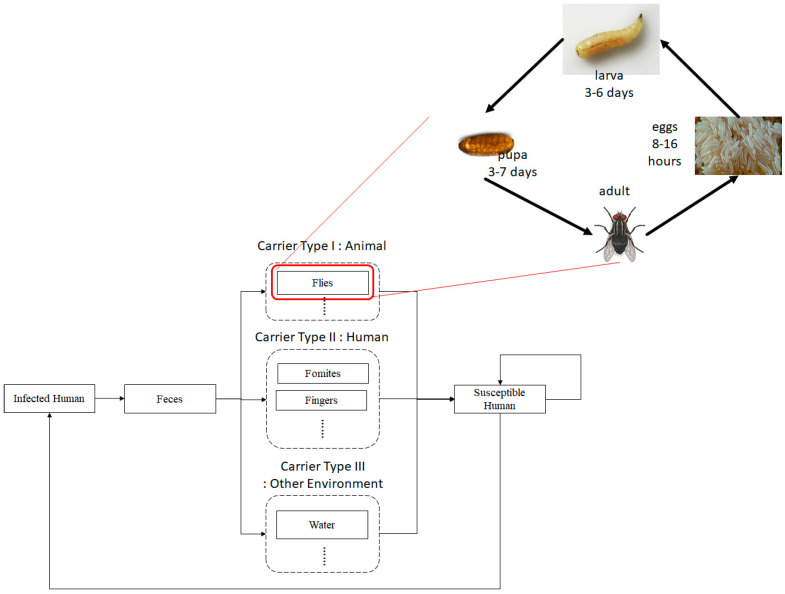
Flow diagram of disease transmission through multiple carriers and a life cycle of a fly as a principal disease carrier.

Figure 1 shows a flow diagram of diarrhea transmission through multiple carriers, including humans, flies and other environmental carriers. The system contains humans’ and flies’ epidemiologic systems, which are transformed into both (human and flies) compartmental models. As shown in Figure 1, the flies system shows the lifecycle of flies from egg stage $\left(E_{f}\right)$, larva $\left(L_{f}\right)$, pupa $\left(U_{f}\right)$, until adult flies, which divides into susceptible $\left(S_{f}\right)$, and carrier flies $\left(C_{f}\right)$. A carrier means an agent that carries a pathogen causing the disease but it does not show any symptoms of illness.

**Figure 2:**
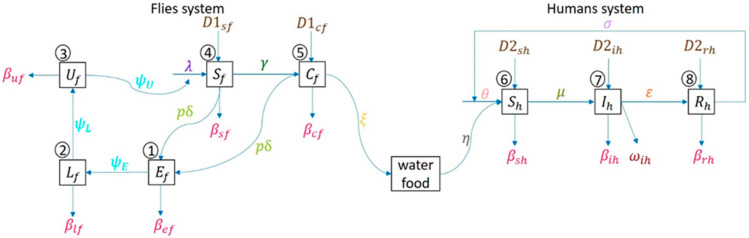
Network model of flies’ life cycle (egg-larva-pupa-adult) and human as susceptible-infected-recovered in compartmental model.

The human system follows susceptible human $\left(S_{h}\right)$, infectious $\left(I_{h}\right)$, recovered $\left(R_{h}\right)$, and back to susceptible again, since no one has immunity against diarrhea. Figure 2 shows an epidemiologic network where human can be infected by the disease by consuming contaminated food or water due to carrier flies laying pathogens on it.

As shown in Figure 2, a set of mathematical models are derived from the fly-human compartmental model. The model parameters in Figure 2 are provided in Table 1.

Table 2 shows the differential equation-based transition models with respect to the lifecycle of the fly and the epidemiologic processes of a human. Humans are classified with the Susceptible-Infected-Recovered-Susceptible (SIRS) reaction-diffusion compartmental model. The ordinary differential equations (ODE) are generated based on each of states in the network. The time unit is “day” in this research.

The solutions of the compartment models, as shown in Table 2, have to be non-negative (positiveness) and exist (boundedness). The below conditions show the positiveness and boundedness of the compartment model.

Let the initial dataset be $S_{h}\left(0\right)>0,\;I_{h}\left(0\right)>0,\;S_{f}\left(0\right)>0,\;C_{f}\left(0\right)>0,\;E_{f}\left(0\right)>0$ and $(L_{f}\left(0\right),{\;U}_{f}\left(0\right),{\;R}_{h}\left(0\right))\in ∐$. ∐ is positive and bounded interval for all time t>0.

Consider the Inequality (1) at time t.

(1) $$\frac{{\mathrm{dE}}_{f}}{\mathrm{dt}}\geq -\left(\beta_{\mathrm{ef}}+\psi_{e}\right)E_{f}$$

(2) $$\int \frac{{\mathrm{dE}}_{f}}{E_{f}}\geq -\int (\beta_{\mathrm{ef}}+\psi_{e})d\left(t\right)$$

(3) $$E_{f}\left(t\right)\geq E_{f}\left(0\right)e^{-\int (\beta_{\mathrm{ef}}+\psi_{e})d\left(t\right)}\geq 0$$

Inequality (1) proves that the solution set is positive for all time t>0. The same rule can be applied for Inequality (2) until Inequality (6) at time t.

(4) $$\frac{{\mathrm{dL}}_{f}}{\mathrm{dt}}\geq -\left(\beta_{\mathrm{lf}}+\psi_{l}\right)L_{f}$$

(5) $$\int \frac{{\mathrm{dL}}_{f}}{L_{f}}\geq -\int (\beta_{\mathrm{lf}}+\psi_{l})d\left(t\right)$$

(6) $$L_{f}\left(t\right)\geq L_{f}\left(0\right)e^{-\int (\beta_{\mathrm{lf}}+\psi_{l})d\left(t\right)}\geq 0$$

## 3. Epidemic Model Dynamics

The dynamics of the models provided in Table 2 depend on the basic reproduction number, which is defined as the average number of secondary infections of an infectious human [18,19].

The basic reproduction number is denoted by $R_{0}$ as the number of secondary infections caused by an infected individual. If the value of $R_{0}<1$, then the disease dies out. While the value of $R_{0}>1$, then the number of infectious individuals increases and the disease invades the population. Let $x={\left(x_{1},x_{2},\ldots ,x_{n}\right)}^{T}$ be the number of individuals in each compartment where the first m<n compartments contain infected individuals. The disease-free equilibrium (DFE) is given by (S,E,I,R) = ($S_{0},0,0,0$). The terms S, E, I and R mean Susceptible, Exposed, Infected, and Recovered, respectively. In this model dynamics, it is assumed that the DFE exists and is stable in the absence of disease. Consider those equations written in the form $\frac{{\mathrm{dx}}_{i}}{\mathrm{dt}}=F_{i\left(x\right)}–V_{i\left(x\right)}$ for i=1,2,…,m where, $F_{i\left(x\right)}$ is the rate of appearance of new infections in compartment i and $V_{i\left(x\right)}$ is the rate of other transitions between compartment i and other infected compartments. F ($F=\left[\frac{\partial F_{i\left(x_{0}\right)}}{\partial x_{j}}\right]$) is entry wise non-negative and V ($V=\left[\frac{\partial \mathrm{Vi}\left(x_{0}\right)}{\partial x_{j}}\right]$) is a non-singular matrix for 1≤i,j≤m. F(X,Y) denotes a vector of new infection rates (flows from X to Y) and V(X,Y) is a vector of all other rates. For each compartment, an inflow in V is negative and an outflow in V is positive. It is assumed that F(0,Y)=0 and V(0,Y)=0. ${\mathrm{FV}}^{-1}$ is called the next generation matrix where the spectral radius of it is equal to $R_{0}$, which is the largest eigenvalue of ${\mathrm{FV}}^{-1}$. ${\mathrm{FV}}^{-1}$ is derived using Equations (7)–(10).

(7) $$F=\left[\frac{\partial {\mu S}_{h}I}{\partial I}\right]=\left[{\mu S}_{h}\right]$$

(8) $$V=\left[\frac{\partial \left(\beta_{\mathrm{ih}}+\omega_{\mathrm{ih}}+\varepsilon +D2_{\mathrm{ih}}\right)I_{h}-\partial {\mu S}_{h}}{\partial I_{h}}\right]$$

(9) $$V^{-1}=\frac{1}{\beta_{\mathrm{ih}}+\omega_{\mathrm{ih}}+\varepsilon +D2_{\mathrm{ih}}}$$

(10) $${\mathrm{FV}}^{-1}=\left[\frac{{\mu S}_{h}}{\beta_{\mathrm{ih}}+\omega_{\mathrm{ih}}+\varepsilon +D2_{\mathrm{ih}}}\right]$$

Then, $R_{0}$ is determined as the basic reproduction number using ${\mathrm{FV}}^{-1}$.

(11) $$R_{0}=\frac{{\mu S}_{h}}{\beta_{\mathrm{ih}}+\omega_{\mathrm{ih}}+\varepsilon +D2_{\mathrm{ih}}}$$

**Figure 3:**
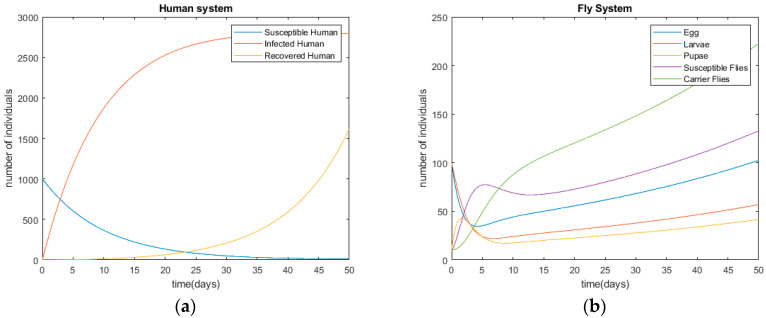
Diseases’ infection trends of diarrhea without control methods. (**a**) Human system; (**b**) fly system.

After modeling the infection processes of diarrhea provided in Section 2 and Section 3, the derived differential equations are solved with the initial conditions of $E_{f}=10$; $L_{f}=10$; $U_{f}=10$; $S_{f}=10$; $C_{f}=10$; $S_{h}=1000$; $I_{h}=1$; $R_{h}=0$. The initial parameters are supposed with real-world examples. Figure 3 shows the disease’s infection simulations without any control method, in terms of humans’ and flies’ status. The simulation is performed using Matlab©. According to Figure 3, the disease starts with an outbreak at *t =* 0 and, the number of infected people is around 2700 at *t =* 20. The time unit is day in this research.

The provided ODE-based compartment model is used to check the epidemiologic processes of diarrhea and the effectiveness of control methods, which are provided in the following section.

## 4. Effective Control Framework Considering Multiple Disease Carriers

In this section, the previous fundamental infection model is extended with several control strategies on the spread of the disease. The term “effectiveness” is evaluated with the blocks of additional infections and a cost-effective concept. This consideration may help to block the spread of SARS-2 or COIVID-19. The cost-effective criteria are determined using Pontryagin’s [20] maximum principle on five variations of control methods. Pontryagin [20,21] introduced the idea of adjoint functions which has a similar purpose as Lagrange multipliers, to append the differential equation to the objective function.

This section focuses on effective control frameworks to prevent additional infections of humans. In order to control additional spreads of diarrhea, five control methods are considered: (1) elimination of fly’s breeding site, (2) sanitation-related investment, (3) installation of UV light traps for killing flies, (4) good personal and food hygiene, and (5) water purification. In general, these methods intend to prevent additional infections among humans or to remove one of primary disease carriers—flies.

As the first control method, “Elimination of flies’ breeding site” is considered. As flies feed on garbage, food waste, and animal feces, therefore one way to prevent additional infections is to keep the places clean, and to spray pesticide on prone areas. This intervention could reduce or even eliminate the maturation rate of fly eggs, larva, and pupa, which eventually impacts the number of adult flies. Let α denote the level of a breeding site elimination strategy (0≤α(t)≤1). The effect of breeding site elimination will decrease the maturation rate of eggs, larvae, and pupae, which is modelled as $\alpha \cdot E_{f},\;\alpha \cdot L_{f}\;$and $\alpha \cdot U_{f}$, respectively.

The second control option is sanitation efforts [22], which include the sterilization of cooking utensils, washing, and drinking water by boiling it properly. The parameter π(t) (0≤π(t)≤1) denotes the level of sanitation strategy. The effect of sanitation is to decrease the number of pathogens, which is modelled as a reduction in the rate of the carrier fly population by the term $\pi \cdot C_{f}$.

The third control method is to install light traps. When flies see ultra-violet (UV) light, they are naturally lured in the direction of the source of the UV rays. This strategy is more desirable than spray for indoor fly control as it keeps the surroundings clean. ϑ (0≤ϑ(t)≤1) denotes the effectiveness of the installation of UV light trap strategy. The effect of this effort is to decrease the number of susceptible and carrier flies by the terms $\vartheta \cdot S_{f}$ and $\vartheta \cdot C_{f}$.

The next consideration is to keep good personal and food hygiene. Good personal and food hygiene can be defined as handling, preparing, and storing food in a way that reduces the risk of becoming contaminated. Let ρ (0≤ρ(t)≤1) be the good personal and food hygiene effort. The effect of this effort is to suppress the number of infected people by the term $\rho \cdot I_{h}$.

The final option is to purify water. Chlorine is often a choice for water purification, since it effectively inactivates the bacteria causing diarrhea, leaves residual protection, has low cost and is easy to transport and use. There are two chlorine-based options [23] used in diarrhea outbreaks, such as tablets and liquid. τ (0≤τ(t)≤1) denotes the level of water purification strategy. The effect of water purification is to inactivate pathogens, which is modelled as a reduction in the disease transmission rate by the term $\tau \cdot I_{h}.$ Table 3 explains each control parameter for each control option.

Based on the control parameters shown in Table 3, the initial transmission models are modified with additional terms to Equations (12)–(17). The additional term is underlined in each equation.

(12) $$\frac{{\mathrm{dE}}_{f}}{\mathrm{dt}}=p.S_{f}.\delta +p.C_{f}.\delta -\beta_{\mathrm{ef}}.E_{f}-\psi_{e}.E_{f}-\underline{\alpha .E_{f}}$$

(13) $$\frac{{\mathrm{dL}}_{f}}{\mathrm{dt}}=\psi_{e}.E_{f}-\beta_{\mathrm{lf}}.L_{f}-\psi_{l}.L_{f}-\underline{\alpha .L_{f}}$$

(14) $$\frac{{\mathrm{dU}}_{f}}{\mathrm{dt}}=\psi_{l}.L_{f}-\beta_{\mathrm{uf}}.U_{f}-\psi_{u}.U_{f}-\underline{\alpha .U_{f}}$$

(15) $$\frac{{\mathrm{dS}}_{f}}{\mathrm{dt}}=\lambda .S_{f}+\psi_{u}.U_{f}-\beta_{\mathrm{sf}}.S_{f}-\gamma .S_{f}+D1_{\mathrm{sf}}.S_{f}\underline{-\pi .S_{f}-\vartheta .S_{f}}$$

(16) $$\frac{{\mathrm{dC}}_{f}}{\mathrm{dt}}=\gamma .S_{f}-\beta_{\mathrm{cf}}.C_{f}+D1_{\mathrm{cf}}.C_{f}\underline{-\;\pi .C_{f}-\vartheta .C_{f}}$$

(17) $$\frac{{\mathrm{dI}}_{h}}{\mathrm{dt}}=\mu .S_{h}-\beta_{\mathrm{ih}}.I_{h}-\omega_{\mathrm{ih}}I_{h}-\varepsilon .I_{h}+D2_{\mathrm{ih}}.I_{h}\underline{-\rho .I_{h}-\tau .I_{h}}$$

In this research, an effective control model is considered as a cost-effective control method within a controllable infection size. The objective function that aims to minimize cost in the control strategy J={α, π, ϑ, ρ, τ} is obtained by Equation (18). In Equation (18), the parameter $W_{1}$ denotes the cost for elimination of breeding site, $W_{2}$ is the cost for sanitation-based works, $W_{3}$ is the cost for installing UV light trap, $W_{4}$ is the cost for isolation of infected person, and $W_{5}$ is the cost of water purification. Each $C_{i,i\in \left[1,5\right]}$ denotes each control parameter-based cost.

(18) $$\begin{array}{ll} C_{\mathrm{stf}}\left(T\right)=\min\int_{0}^{T} & [W_{1}\cdot \alpha \left(t\right)\cdot K_{1}+W_{2}\cdot \pi \left(t\right)\cdot K_{2}+W_{3}\cdot \vartheta \left(t\right)\cdot K_{2}+W_{4}\cdot \rho \left(t\right)\cdot I_{h}\left(t\right)\\ & +W_{4}\cdot \rho \left(t\right)\cdot I_{h}\left(t\right)+W_{5}\cdot \tau \left(t\right)\cdot I_{h}\left(t\right)+\frac{1}{2}\cdot K_{3}]\mathrm{dt} \end{array}$$

where $K_{1}=E_{f}\left(t\right)+L_{f}\left(t\right)+U_{f}\left(t\right)$, $K_{2}=S_{f}\left(t\right)+C_{f}\left(t\right)$, and $K_{3}=C_{1}\alpha^{2}+C_{2}\pi^{2}+C_{3}\vartheta^{2}+C_{4}\rho^{2}+C_{5}{\;\tau }^{2}$.

Due to the fact that the cost function is nonlinear with the infection trends, time and other conditions, the mathematical programming belongs to the differential equation-based nonlinear mathematical programming. When denoting the prices associated with their respective classes by $\phi_{E_{f}},\;\phi_{L_{f}},\;\phi_{U_{f}},\;\phi_{S_{f}},\;\phi_{C_{f}},\;\phi_{S_{h}},\;\phi_{I_{h}}\;and\;\phi_{R_{h}}$, the nonlinear mathematical programming is solved using Pontryagin’s maximum principle and the derivation of necessary conditions. As explained in [24,25], the existence of an optimal control is a sequence of the convexity of the integrand J with respect to α, π, ϑ, ρ, τ; a priori boundedness of the state variables, and the Lipschitz property of the state system with respect to the state variables. The differential equations are obtained by differentiating the Hamiltonian function, as shown in Appendix A. Finally, optimal control values are obtained as shown in Appendix B.

(19) $$\alpha^{c}=\left[\left(E_{f}^{*}\phi_{E_{f}}+L_{f}^{*}\phi_{L_{f}}+U_{f}^{*}\phi_{U_{f}}\right)-W_{1}\left(E_{f}^{*}+L_{f}^{*}+U_{f}^{*}\right)\right]/C_{1}$$

(20) $$\pi^{c}=\left[\left(S_{f}^{*}\phi_{S_{f}}+C_{f}^{*}\phi_{C_{f}}\right)-W_{2}\left(S_{f}^{*}+C_{f}^{*}\right)\right]/C_{2}$$

(21) $$\vartheta^{c}=\left[\left(S_{f}^{*}\phi_{S_{f}}+C_{f}^{*}\phi_{C_{f}}\right)-W_{3}\left(S_{f}^{*}+C_{f}^{*}\right)\right]/C_{3}$$

(22) $$\rho^{c}=\left[I_{h}^{*}\phi_{I_{h}}-W_{4}I_{h}^{*}\right]/C_{4}$$

(23) $$\tau^{c}=\left[I_{h}^{*}\phi_{I_{h}}-W_{5}I_{h}^{*}\right]/C_{5}$$

Therefore, it can be concluded by the standard control arguments involving the bounds on the controls in Table 4 as follows:

## 5. Simulation and Analysis of Control Model Considering Multiple Disease Carriers

With the provided optimal control frameworks, numerical simulations are performed using the parameter values given in Table 1 and Table 3. Several control scenarios are considered with combinations of intervention strategies for non-adult flies, adult flies, and infected humans, as summarized in Table 5. Table 5 shows the highest number of flies and humans using 50 days’ of simulation.

**Figure 4:**
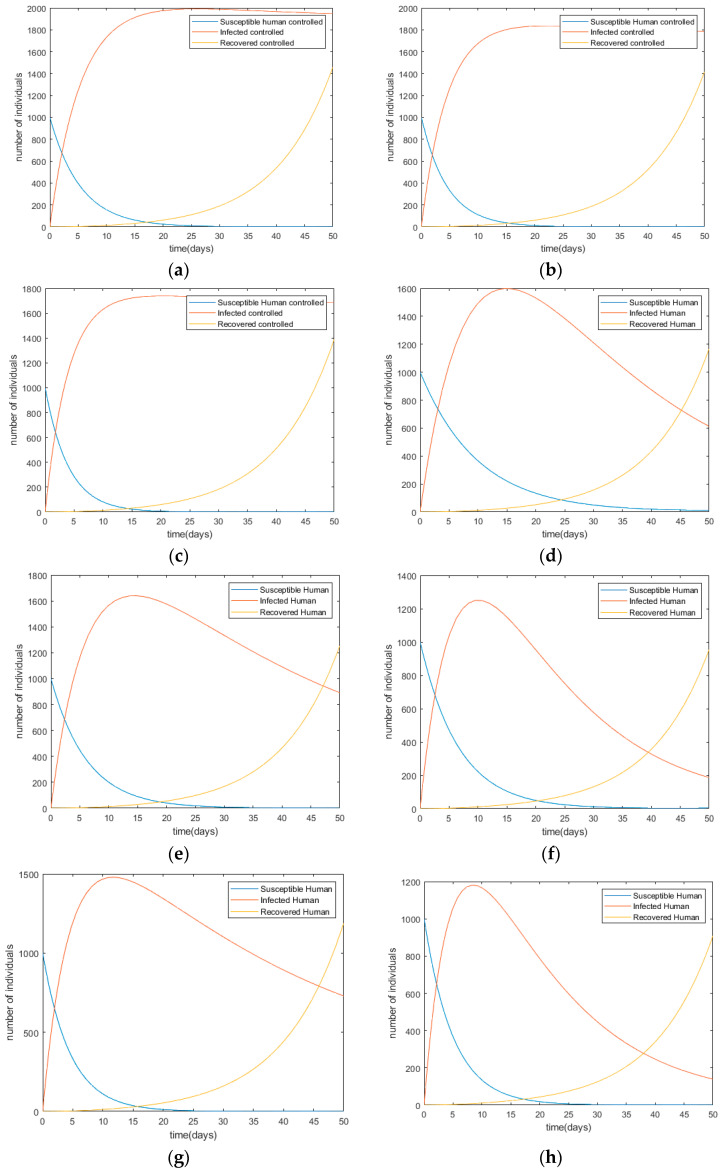

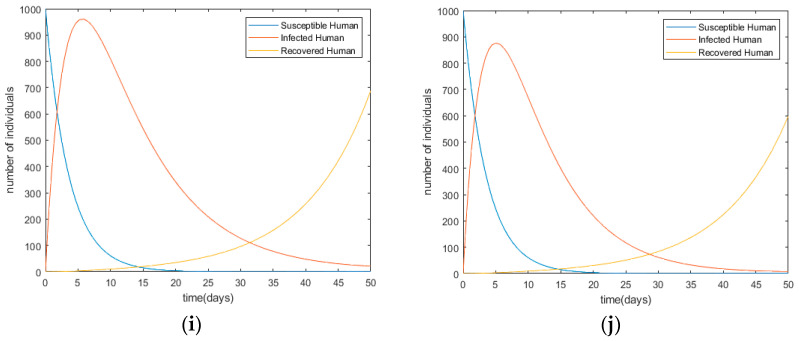
Changes in infection trends toward the human population with each control scenario (**a**) Scenario I; (**b**) Scenario II; (**c**) Scenario III; (**d**) Scenario IV; (**e**) Scenario V; (**f**) Scenario VI; (**g**) Scenario VII; (**h**) Scenario VIII; (**i**) Scenario IX; (**j**) Scenario X.

Each control is applied and is combined with more than one control to examine the impact on the human population. The simulation results are shown in Figure 4.

Table 6 shows the assumed control costs and medical treatment costs. The costs are assigned with the consideration of existing relevant literatures [26,27,28,29,30].

After comparing all of those ten scenarios, the most effective strategy is considered Scenario X, which combines all control method under the assumptions. Table 7 shows the simulation result for each control scenario I to scenario X.

In terms of cost and the number of infected persons, Scenario X shows the best performance. This indicates that the overall control methods are required for effective disease control and their portions influence its performance. It also denotes the significant reduction in number of infected people from 2795 to 877 as a result of the decreasing number of disease carriers. It means there is a 65.7% reduction in the number of infected people compared to the simulation without applying any control.

The provided framework can be used to check the effectiveness with different assumptions and numerical simulations considering multiple disease carriers.

## 6. Conclusions

This paper presents the ordinary differential equation-based epidemiologic models to understand diarrhea’s infection dynamics and with the consideration of multiple carriers. Numerical simulation and mathematical analyses were performed to identify the relationship and the status of flies and humans when the disease outbreaks.

Then, each control strategy is applied, as shown in Scenarios I to V, and is combined in some combinations of these strategies to obtain the optimal condition, as shown in Scenarios VI to X. Under a certain simulation scenario, Scenario X, which combines all control methods, is proven to be the most effective strategy in reducing both of the number of flies and the infected population, as it minimizes the spread of the disease.

In the future, further study about broader conditions such as the effect of temperature on the transmission of a disease is required, as the population of housefly reaches its peak when the temperature is warmer.

The proposed framework is considered an effective disease control considering multiple carriers with epidemiologic dynamics and the relevant parameters.

## Figures and Tables

**Table 1:** Notations of variables and parameters.

Symbol	Description	Initial Values
**Variables**		
E_f_	The number of eggs of flies	100
L_f_	The number of larvae of flies	100
U_f_	The number of pupae of flies	10
S_f_	The number of susceptible flies	10
C_f_	The number of carrier fly	10
S_h_	The number of susceptible humans	1000
I_h_	The number of infected humans	1
R_h_	The number of recovered humans	0
**Parameters**		
λ	An influx rate of susceptible flies	0.1
γ	A rate of susceptible flies to become carrier	0.2
p	Probability of female fly	0.5
$\psi_{e}$	Average maturation rate from egg to larva	0.4
$\psi_{l}$	Average maturation rate from larva to pupa	0.6
$\psi_{u}$	Average maturation rate from pupa to adult fly	0.7
$\beta_{\mathrm{ef}}$	Natural death rate of eggs	0.1
$\beta_{\mathrm{lf}}$	Natural death rate of larvae	0.1
$\beta_{\mathrm{uf}}$	Natural death rate of pupae	0.1
$\beta_{\mathrm{sf}}$	Natural death rate of susceptible flies	0.1
$\beta_{\mathrm{cf}}$	Natural death rate of carrier flies	0.1
δ	An oviposit rate of adult female flies	0.3
$D1_{\mathrm{sf}}$	Diffusion parameter among susceptible flies	0.001
$D1_{\mathrm{cf}}$	Diffusion parameter among carrier flies	0.001
ξ	Carrier fly’s laying rate of pathogen on water or food	0.6
θ	Influx rate of susceptible humans	0.1
μ	Rate from “susceptible” status to “infected” status in humans	0.3
ε	Rate from “infected” status to “recovered” status in humans	0.0008
σ	Rate from “recovered” status to “susceptible” status in humans	0.001
$\beta_{\mathrm{sh}}$	Natural death rate of susceptible humans	0.0008
$\beta_{\mathrm{ih}}.$	Natural death rate of infected humans	0.0008
$\beta_{\mathrm{rh}}$	Natural death rate of recovered humans	0.0008
$D2_{\mathrm{sh}}$	Diffusion parameter among susceptible humans	0.1
$D2_{\mathrm{ih}}$	Diffusion parameter among infected humans	0.3
$D2_{\mathrm{rh}}$	Diffusion parameter among recovered humans	0.1
η	Rate of contaminated water or food to be consumed by susceptible humans	0.5
$\omega_{\mathrm{ih}}$	Disease-induced death rate of infected humans	0.3

**Table 2:** Ordinary differential equations (ODE) considering both the fly and the human system.

Compartment	ODE
$E_{f}$	$\frac{{\mathrm{dE}}_{f}}{\mathrm{dt}}=\;p.S_{f}.\delta +p.C_{f}.\delta -\beta_{\mathrm{ef}}.E_{f}-\psi_{e}.E_{f}$
$L_{f}$	$\frac{{\mathrm{dL}}_{f}}{\mathrm{dt}}={\;\psi }_{e}.E_{f}-\beta_{\mathrm{lf}}.L_{f}-\psi_{l}.L_{f}$
$U_{f}$	$\frac{{\mathrm{dU}}_{f}}{\mathrm{dt}}=\psi_{l}.L_{f}-\beta_{\mathrm{uf}}.U_{f}-\psi_{u}.U_{f}$
$S_{f}$	$\frac{{\mathrm{dS}}_{f}}{\mathrm{dt}}=\lambda .S_{f}+\psi_{u}.U_{f}-\beta_{\mathrm{sf}}.S_{f}-\gamma .S_{f}+D1_{\mathrm{sf}}.S_{f}$
$C_{f}$	$\frac{{\mathrm{dC}}_{f}}{\mathrm{dt}}=\gamma .S_{f}-\beta_{\mathrm{cf}}.C_{f}+D1_{\mathrm{cf}}.C_{f}$
$S_{h}$	$\frac{{\mathrm{dS}}_{h}}{\mathrm{dt}}=\;\theta .S_{h}-\beta_{\mathrm{sh}}.S_{h}-\mu .S_{h}+\sigma .R_{h}+D2_{\mathrm{sh}}.S_{h}$
$I_{h}$	$\frac{{\mathrm{dI}}_{h}}{\mathrm{dt}}=\;\mu .S_{h}-\beta_{\mathrm{ih}}.I_{h}-\omega_{\mathrm{ih}}I_{h}-\varepsilon .I_{h}+D2_{\mathrm{ih}}.I_{h}$
$R_{h}$	$\frac{{\mathrm{dR}}_{h}}{\mathrm{dt}}=\;\varepsilon .I_{h}-\beta_{\mathrm{rh}}.R_{h}-\sigma .R_{h}+D2_{\mathrm{rh}}.R_{h}$

μ=ξ·η.

**Table 3:** Control parameters.

Symbol	Description	Initial Values
**Control Parameters**		
α	Effective control using eliminations of fly’s breeding site	0.03
π	Effective rate using sanitation methods	0.1
ϑ	Effective rate using installation of UV light trap	0.04
ρ	Effective rate using good personal and food hygiene	0.01
τ	Effective rate using water purification	0.02

**Table 4:** The optimal control parameter of each strategy.

Control Parameter	The Optimal Value of Control Parameter
$\alpha^{*}$	$\{\begin{array}{c} 0\\ \alpha^{c}\\ 1 \end{array}$ $\begin{array}{c} \mathrm{if}\\ \mathrm{if}\\ \mathrm{if} \end{array}$ $\begin{array}{c} \alpha^{c}\leq 0\\ 0<\alpha^{c}<1\\ \alpha^{c}\geq 1 \end{array}$
$\pi^{*}$	$\{\begin{array}{c} 0\\ \pi^{c}\\ 1 \end{array}$ $\begin{array}{c} \mathrm{if}\\ \mathrm{if}\\ \mathrm{if} \end{array}$ $\begin{array}{c} \pi^{c}\leq 0\\ 0<\pi^{c}<1\\ \pi^{c}\geq 1 \end{array}$
$\vartheta^{*}$	$\{\begin{array}{c} 0\\ \vartheta^{c}\\ 1 \end{array}$ $\begin{array}{c} \mathrm{if}\\ \mathrm{if}\\ \mathrm{if} \end{array}$ $\begin{array}{c} \vartheta^{c}\leq 0\\ 0<\vartheta^{c}<1\\ \vartheta^{c}\geq 1 \end{array}$
$\rho^{*}$	$\{\begin{array}{c} 0\\ \rho^{c}\\ 1 \end{array}$ $\begin{array}{c} \mathrm{if}\\ \mathrm{if}\\ \mathrm{if} \end{array}$ $\begin{array}{c} \rho^{c}\leq 0\\ 0<\rho^{c}<1\\ \rho^{c}\geq 1 \end{array}$
$\tau^{*}$	$\{\begin{array}{c} 0\\ \tau^{c}\\ 1 \end{array}$ $\begin{array}{c} \mathrm{if}\\ \mathrm{if}\\ \mathrm{if} \end{array}$ $\begin{array}{c} \tau^{c}\leq 0\\ 0<\tau^{c}<1\\ \tau^{c}\geq 1 \end{array}$

A *: optimal value of parameter of A.

**Table 5:** Scenarios with combinations of control strategies.

Scena-Rio	Strategy\Control Parameters	Egg Flies	Larva	Pupa	Susceptible Flies	Carrier Flies	Infected Human
-	Initial condition (without controls)	126	70	51	163	273	2795
I	Elimination of breeding site (α=0.03)	5	0	0	133	223	1991
II	Sanitation (π=0.1)	103	57	42	27	4	1837
III	Installation of UV light trap (ϑ=0.04)	69	38	28	4	33	1739
IV	Good personal and food hygiene (ρ=0.01)	103	57	42	133	223	1599
V	Water purification (τ=0.02)	103	57	42	133	223	1641
VI	Combination of I, II and IV (α=0.03,π=0.1,ρ=0.01)	50	13	10	30	50	1251
VII	Combination of I, II and V (α=0.03,π=0.1,τ=0.02)	23	13	10	30	50	1480
VIII	Combination of I, III and IV (α=0.03,ϑ=0.04,ρ=0.01)	11	7	5	15	21	1182
IX	Combination of I, III and V (α=0.03,ϑ=0.04,τ=0.02)	17	10	7	22	36	961
X	Combination of I-V (α=0.03,π=0.1,ϑ=0.04,ρ=0.01,τ=0.02)	0	0	0	0	0	877

**Table 6:** Cost values for control simulations.

Parameter	Unit Cost ($)
**Control costs**	
Eliminations of fly’s breeding site	100
Sanitation methods	60
Installation of UV light trap	240
Good personal and food hygiene	1138
Water purification	0.46
**Medical treatment cost**	
Hospitalization	207.7

**Table 7:** Result of control scenario.

Control Scenario	Relevant Figure	Cost ($)	Results
I	Figure 4a	413,632	- Maturation rates of eggs, larva, and pupa are decreased since they died before even matured. - The number of eggs of fly, larva, and pupa vanish at *t =* 42 while the number of infected humans reaches its peak at *t =* 26 with 1991 people and then started to decrease compared with the initial simulation. - An outbreak at *t =* 23 along with the increasing number of flies.
II	Figure 4b	381,605	- Sanitation aim to increase the death rate of adult flies, both the susceptible and carrier flies. - Applying this intervention impacts to the number of susceptible and carrier flies significantly decrease to 27 and 4 respectively while for initial simulation, there are 163 susceptible and 273 carrier flies. - At *t =* 24, the number of infected human starts decreasing after reaching its maximum with 1837 people get the disease.
III	Figure 4c	361,430	- This intervention will trap and catch the adult flies which means the death rate of adult flies is increased. - Thus, the population number of susceptible and carrier flies reaches its lowest at *t =* 50 with 4 and 33 flies. - The highest number of infected humans is 1739 people which is lower that Strategy II.
IV	Figure 4d	333,250	- By keeping good personal and food hygiene, it does not give opportunity for the pathogen which cause the disease to spread among humans. - Applying this control leads to almost half of reduction number of infected humans at *t =* 13 with 1373 people compared to the initial simulation
V	Figure 4e	340,836	- Ensure every healthy people to only consume hygiene water is like cutting the chain of the disease to be spreading in the environment. - It will affect the reduction rate of contaminated water to be consumed by human. Therefore, the number of infected people will decrease at *t =* 14 after experiencing its peak with 1641 people.
VI	Figure 4f	261,131	- Combination of these strategies will tackle each of non-adult flies (eggs, larva, pupa), adult flies, and infected human population. - At time *t =* 70, the number of infected people is almost zero meanwhile the disease infected the highest number 1251 people at *t =* 10 before the transmission becomes slower after that.
VII	Figure 4g	307,556	- After performing this scenario, the number of non-adult flies has much reduction at *t =* 48 which is good for indicating the decreasing of the adult flies’ population as well. - The largest population of infected people is 1.480 at *t =* 12 then the infection subsided.
VIII	Figure 4h	246,979	- Combination of these strategies could lead to the reduction number of all individual, where at *t =* 36, the population of flies is almost zero and the highest number of infected humans is 1182 at *t =* 9.
IX	Figure 4i	199,940	- These combinations provide a good result in decreasing the maturation rate of non-adult flies and increasing the death rate of adult flies which affects to the reduction rate of susceptible status becomes infected status in human. At *t =* 7, the number of infected humans is 961 people but the population becomes disease-free at *t =* 50 since no one is infected.
X	Figure 4j	183,691	- The population of non-adult and adult flies are zero at *t =* 27 meanwhile the highest number of infected people is 877 before reaching the diseases free at *t =* 43. - Combination of all strategies surely give the best result in terms of number of each individual. - In addition, the scenario achieves the lowest cost among all scenarios.

## Appendix A

Consider the objective function J(α, π, ϑ, ρ, τ) to investigate the optimal strategy needed to control the spreading of diarrhea provided in Equation, where $A_{1},\ldots ,A_{8}$ represent the weight constants of the eggs, larva, pupa, susceptible flies, carrier flies, susceptible human, infected human, and recovered human.

$$\begin{array}{ll} J\left(\alpha ,\;\pi ,\;\vartheta ,\;\rho ,\;\tau \right)= & \int_{0}^{T_{f}}[A_{1}E_{f}+A_{2}L_{f}+A_{3}U_{f}+A_{4}S_{f}+A_{5}C_{f}+A_{6}S_{h}+A_{7}I_{h}+A_{8}R_{h}\\ & +\frac{1}{2}\left(C_{1}\alpha^{2}+C_{2}\pi^{2}+C_{3}\vartheta^{2}+C_{4}\rho^{2}+C_{5}{\;\tau }^{2}\right)]\mathrm{dt} \end{array}$$

The Hamiltonian H is provided with J(α, π, ϑ, ρ, τ) and the ODEs, where $\phi_{E_{f},\;}\phi_{L_{f}},\phi_{U_{f}},\;\phi_{S_{f}},\;\phi_{C_{f},\;}\phi_{S_{h}},\;\phi_{I_{h}},\;\phi_{R_{h}},\phi_{C_{\mathrm{stf}}}$ are the co-state variables given by the system:

$$H=\left[A_{1}E_{f}+A_{2}L_{f}+A_{3}U_{f}+A_{4}S_{f}+A_{5}C_{f}+A_{6}S_{h}+A_{7}I_{h}+A_{8}R_{h}+\frac{1}{2}\left(C_{1}\alpha^{2}+C_{2}\pi^{2}+C_{3}\vartheta^{2}+C_{4}\rho^{2}+C_{5}{\;\tau }^{2}\right)\right]\;+\phi_{E_{f}}\left[p.S_{f}\left(t\right).\delta +p.C_{f}\left(t\right).\delta -\beta_{\mathrm{ef}}.E_{f}\left(t\right)-\psi_{e}.E_{f}\left(t\right)-\alpha .E_{f}\left(t\right)\right]\;+\phi_{L_{f}}\left[\psi_{e}.E_{f}\left(t\right)-\beta_{\mathrm{lf}}.L_{f}\left(t\right)-\psi_{l}.L_{f}\left(t\right)-\alpha .L_{f}\left(t\right)\right]\;+\phi_{U_{f}}\left[\psi_{l}.L_{f}\left(t\right)-\beta_{\mathrm{uf}}.U_{f}\left(t\right)-\psi_{u}.U_{f}\left(t\right)-\alpha .U_{f}\left(t\right)\right]\;+\phi_{S_{f}}\left[\lambda .S_{f}\left(t\right)+\psi_{u}\left(t\right).U_{f}\left(t\right)-\beta_{\mathrm{sf}}.S_{f}\left(t\right)-\gamma .S_{f}\left(t\right)+D1_{\mathrm{sf}}.S_{f}\left(t\right)-\pi .S_{f}\left(t\right)-\vartheta .S_{f}\left(t\right)\right]\;+\phi_{C_{f}}\left[\gamma .S_{f}\left(t\right)-\beta_{\mathrm{cf}}.C_{f}\left(t\right)+D1_{\mathrm{cf}}.C_{f}\left(t\right)-\;\pi .C_{f}\left(t\right)-\vartheta .C_{f}\left(t\right)\right]\;+\phi_{S_{h}}\left[\theta .S_{h}\left(t\right)-\beta_{\mathrm{sh}}.S_{h}\left(t\right)-\mu .S_{h}\left(t\right)+\sigma .R_{h}\left(t\right)+D2_{\mathrm{sh}}.S_{h}\left(t\right)\right]\;+\phi_{I_{h}}\left[\mu .S_{h}\left(t\right)-\beta_{\mathrm{ih}}.I_{h}\left(t\right)-\omega_{\mathrm{ih}}I_{h}\left(t\right)-\varepsilon .I_{h}\left(t\right)+D2_{\mathrm{ih}}.I_{h}\left(t\right)-\rho .I_{h}\left(t\right)-\tau .I_{h}\left(t\right)\right]\;+\phi_{R_{h}}\left[\varepsilon .I_{h}\left(t\right)-\beta_{\mathrm{rh}}.R_{h}\left(t\right)-\sigma .R_{h}\left(t\right)+D2_{\mathrm{rh}}.R_{h}\left(t\right)\right]\;+\phi_{C_{\mathrm{stf}}}\left[W_{1}{\alpha E}_{f}\left(t\right)L_{f}\left(t\right)U_{f}\left(t\right)+W_{2}{\pi S}_{f}\left(t\right)C_{f}\left(t\right)+W_{3}\;\vartheta S_{f}\left(t\right)C_{f}\left(t\right)+W_{4}{\rho I}_{h}\left(t\right)+W_{5}{\tau I}_{h}\left(t\right)\right]$$

## Appendix B

Optimal control values are obtained using the Hamiltonian function shown in Appendix A. Then, the nonlinear mathematical programming is solved using Pontryagin’s Maximum Principle and the derivation of necessary conditions.

$$\frac{\partial H}{\partial \alpha }=\alpha^{c}C_{1}+\left[-\left(E_{f}^{*}\phi_{E_{f}}+L_{f}^{*}\phi_{L_{f}}+U_{f}^{*}\phi_{U_{f}}\right)+W_{1}\left(E_{f}^{*}+L_{f}^{*}+U_{f}^{*}\right)\right]\;\frac{\partial H}{\partial \pi }=\pi^{c}C_{2}+\left[-\left(S_{f}^{*}\phi_{S_{f}}+C_{f}^{*}\phi_{C_{f}}\right)+W_{2}\left(S_{f}^{*}+C_{f}^{*}\right)\right]\;\frac{\partial H}{\partial \vartheta }=\vartheta^{c}C_{3}+\left[-\left(S_{f}^{*}\phi_{S_{f}}+C_{f}^{*}\phi_{C_{f}}\right)+W_{3}\left(S_{f}^{*}+C_{f}^{*}\right)\right]\;\frac{\partial H}{\partial \rho }={\;\rho }^{c}C_{4}+\left[-\left(I_{h}^{*}\phi_{I_{h}}\right)+W_{4}I_{h}^{*}\right]\;\frac{\partial H}{\partial \tau }={\;\tau }^{c}C_{5}+\left[-\left(I_{h}^{*}\phi_{I_{h}}\right)+W_{5}I_{h}^{*}\right]$$

## References

[1] GBD 2016 Causes of Death Collaborators (2017). Global, regional, and national age-sex specific mortality for 264 causes of death, 1980–2016: A systematic analysis for the Global Burden of Disease Study 2016. Lancet.

[2] InformedHealth.org (2016). Cologne, Germany: Institute for Quality and Efficiency in Health Care (IQWiG). 2006. Acute infectious Diarrhea: Common Germs and Routes of Infection. https://www.ncbi.nlm.nih.gov/books/NBK373086/.

[3] Olsen A.R., Hammack T.S. (2000). Isolation of Salmonella spp. from the housefly, *Musca domestica* L., and the dump fly, Hydrotaea aenescens (Wiedemann) (Diptera: Muscidae), at caged-layer houses. J. Food Prot..

[4] Hosseini S.M., Zeyni B., Rastyani S., Jafari R., Shamloo F., Tabar Z.K., Rabestani M.R. (2016). Presence of virulence factors and antibiotic resistances in Enterococcus sp collected from dairy products and meat. Der Pharm. Lett..

[5] Tsagaan A., Kanuka I., Okado K. (2015). Study of pathogenic bacteria detected in fly samples using universal primer-multiplex PCR. Mong. J. Agric. Sci..

[6] Nassiri H., Zarrin M., Veys-Behbahani R., Faramarzi S., Nasiri A. (2015). Isolation and identification of pathogenic filamentous fungi and yeasts from adult house fly (Diptera: Muscidae) captured from the hospital environments in Alivaz city, Southwestern Iran. J. Med. Entomol..

[7] Peffly R.L. (1953). A summary of recent studies on house flies in Egypt. J. Egypt. Public Health Assoc..

[8] Chavasse D.C., Shier R.P., Murphy O.A., Huttly S.R., Cousens S.N., Akhtar A. (1999). Impact of fly control on childhood diarrhea in Pakistan: Community randomized trial. Lancet.

[9] Echeverria P., Harrison B.A., Tirapat C., McFarland A. (1983). Flies as a source of enteric pathogens in a rural village in Thailand. Appl. Environ. Microbiol..

[10] Nash J.T. (1909). House flies as carriers of disease. J. Hyg. (Lond.).

[11] Collinet-Adler S., Babji S., Francis M., Kattula D., Premkumar P.S., Sarkar R., Mohan V.R., Ward H., Kang G., Balraj V. (2015). Environmental Factors Associated with High Fly Densities and Diarrhea in Vellore, India. Appl. Environ. Microbiol..

[12] Das J.K., Hadi Y.B., Salam R.A., Hoda M., Lassi Z.S., Bhutta Z.A. (2018). Fly control to prevent diarrhea in children. Cochrane Database Syst. Rev..

[13] Cohen D., Green M., Block C., Slepon R., Ambar R., Wasserman S.S., Levine M.M. (1991). Reduction of transmission of shigellosis by control of houseflies (Musca domestica). Lancet.

[14] Lindsay D., Stewart W., Watt J. (1953). Effect of fly control on diarrhea in an area of moderate morbidity. Public Health Rep..

[15] Watt J., Lindsay D.R. (1948). Diarrhea control studies; effect of fly control in a high morbidity area. Public Health Rep..

[16] Hartemink N., Vanwambeke S.O., Heesterbeek H., Rogers D., Morley D., Pesson B., Davies C., Mahamdallie S., Ready P. (2011). Integrated mapping of establishment risk for emerging vector-borne infections: A case study of canine leishmaniasis in southwest France. PLoS ONE.

[17] Lenhart S., Workman J.T. (2007). Optimal Control Applied to Biological Models.

[18] Heffernan J., Smith R., Wahl L. (2005). Perspectives on the basic reproductive ratio. J. R. Soc. Interface.

[19] Diekmann O., Heesterbeek J., Metz J.A. (1990). On the definition and the computation of the basic reproduction ratio R_0_ in models for infectious diseases in heterogeneous populations. J. Math. Biol..

[20] Pontryagin L.S. (2018). Mathematical Theory of Optimal Processes.

[21] Getachew T.T., Oluwole D.M., David M. (2017). Modelling and optimal control of pneumonia disease with cost-effective strategies. J. Biol. Dyn..

[22] Cairncross S., Hunt C., Boisson S., Bostoen K., Curtis V., Fung I.C.H., Schmidt W.-P. (2010). Water, sanitation and hygiene for the prevention of diarrhea. Int. J. Epidemiol..

[23] Branz A., Levine M., Lehmann L., Bastable A., Ali S.I., Kadir K., Yates T., Bloom D., Lantagne D. (2017). Chlorination of drinking water in emergencies: A review of knowledge, recommendations for implementation, and research needed. Waterlines.

[24] Okosun K.O., Rachid O., Marcus N. (2013). Optimal control strategies and cost-effectiveness analysis of a malaria model. BioSystems.

[25] Fleming W.H., Rishel R.W. (2017). Deterministic and Stochastic Optimal Control.

[26] How Much Does It Cost to Get Rid Of Flies?. https://www.kompareit.com/homeandgarden/home-services-compare-fly-exterminator-cost.html.

[27] Sandy C., Vivian V. (2006). Water supply, sanitation and hygiene promotion. Disease Control Priorities in Developing Countries.

[28] Golan E.H., Vogel S.J., Frenzen P.D., Ralston K.L. (2000). Tracing the Costs and Benefits of Improvements in Food Safety: The Case of the Hazard Analysis and Critical Control Point Program for Meat and Poultry.

[29] Ephrem T.S., Sirak R., Helmut K., Bezatu M. (2020). Effect of household water treatment with chlorine on diarrhea among children under the age of five years in rural areas of Dire Dawa, eastern Ethiopia: A cluster randomized controlled trial. Infect. Dis. Poverty.

[30] Van M.H., Tuan A.T., Anh D.H., Viet H.N. (2015). Cos of hospitalization for foodborne diarrhea: A case study from Vietnam. J. Korean Med. Sci..

